# Focal cortical dysplasias: modeling pediatric drug-resistant epilepsy using human brain organoids

**DOI:** 10.3389/fncel.2026.1794559

**Published:** 2026-03-10

**Authors:** Sabrina Petralla, Eleonora Crocco, Michela Giustizieri, Luca De Palma, Federico Cremisi, Enrico Cherubini, Nicola Specchio, Antonino Cattaneo, Silvia Marinelli

**Affiliations:** 1 European Brain Research Institute, Rita Levi-Montalcini Foundation, Roma, Italy; 2 Neurology, Epilepsy and Movement Disorders Unit, Bambino Gesù Children's Hospital, IRCCS, Full Member of European Reference Network EpiCARE, Rome, Italy; 3 Laboratorio di Biologia Bio@SNS, Scuola Normale Superiore, Pisa, Italy; 4 Istituto di Biofisica, Consiglio Nazionale delle Ricerche, Pisa, Italy; 5 University Hospitals KU, Leuven, Belgium

**Keywords:** brain organoids, E/I balance, epilepsy, FCD, hiPSC

## Abstract

Epilepsy is a prevalent neurological disorder characterized by recurrent, unprovoked seizures and altered electroencephalographic patterns. This condition is viewed as a malfunctioning of extensive neural networks due to an imbalance of excitatory and inhibitory signals leading neurons to be excessively excitable and to abnormal synchronized electrical activity. Despite the growing number of new antiepileptic drugs, patients suffering from drug-resistant forms of epilepsy do not respond to pharmacological treatment, and the only effective cure remains the neurosurgical resection of the epileptic focus. Nevertheless, several patients fail to achieve seizure freedom after surgical resection. This emphasizes the urgent need for novel human-relevant models to explore the mechanisms underlying drug-refractory forms of epilepsy. While acute and organotypic slices from resected neurological tissue offer a promising method for studying patient-derived brain tissue mechanisms, this technique is limited by its inherently low throughput and challenges in obtaining appropriate control tissue. Recent advances in organoid technology have allowed for the generation of cerebral dorsal/ventral assembloids, which more accurately model the functional connectivity between excitatory and inhibitory neurons and recapitulate key aspects of cortical circuits. This review summarizes current knowledge on the use of human brain organoids and assembloids to model epilepsy, with a particular focus on organoids harboring focal cortical dysplasia-linked mutations. Human brain organoids and assembloids will allow addressing an important question in the field, namely the relative contribution of neurodevelopmental defects vs. those arising at later stages of CNS development. Limitations of this “neuron-only” *in vitro* model and potential ways to include non-neuronal cells will also be discussed. Finally, we highlight recent advances in employing these new powerful platforms for investigating network dysfunctions underlying FCDs, screening potential antiepileptic drug candidates, and developing personalized therapeutic strategies.

## 1 Introduction

Epilepsy is the most common neurological disorder characterized by recurrent seizures caused by abnormal discharges in the brain, often associated with deficits in cognitive functions (Adeyeye et al., 2024). Focal cortical dysplasias (FCDs) are a highly heterogeneous neurodevelopmental malformation and the leading cause of pediatric drug-resistant epilepsy, often accompanied by significant cognitive and behavioral challenges (Palmini and Holthausen, 2013; Blumcke et al., 2017). Like other forms of epilepsy, FCDs are increasingly conceptualized as disorders of large-scale neural network dysfunction, driven by a pathological disruption in the balance between excitatory and inhibitory synaptic signals in selective neuronal circuits (Kim et al., 2025a). This imbalance leads to increased neuronal excitability and aberrant synchronized electrical activity (Xu et al., 2025). FCDs exhibit a wide range of clinical manifestations, with variability in the size of lesion, location, and age of epilepsy onset, often accompanied by differences in seizure severity. Histological examination reveals distinctive features such as cortical disorganization and abnormal cellular elements (Blümcke et al., 2011). Unfortunately, despite the increasing number of new antiepileptic drugs, many patients remain refractory to medical treatment, and in those cases the only effective cure is the neurosurgical resection of the epileptic focus. However, 30–40% of patients fail to achieve seizure freedom after surgical resection (De Tisi et al., 2011; Engel et al., 2013). Evidence from clinical-surgical and electrophysiological investigations in humans, alongside *in vitro* rodent models of cortical dysgenesis, indicates that the epileptogenic zone in FCDs can extend beyond the lesion boundaries detectable by conventional imaging, such as magnetic resonance (Redecker et al., 2000; Cohen-Gadol et al., 2004; Guerrini et al., 2008). Furthermore, subcortical structures such as the thalamus and basal ganglia have been shown to potentially regulate cortical activity and synchronize epileptic discharges (Bernhardt et al., 2012; Výtvarová et al., 2017). This perspective was further strengthened by Pittau et al.'s research, which identified metabolic involvement in remote cortical and subcortical regions in FCD patients, thus promoting the idea of an epileptic network rather than a localized focus (Pittau et al., 2017).

This underscores the urgent need for novel human-relevant models to investigate the mechanisms underlying drug-resistant forms of epilepsy as well as those that remain refractory to surgical resection. A currently promising approach consists in using acute and organotypic slice cultures from neurosurgical resections of epileptic patients (Eugène et al., 2014; Bak et al., 2024). However, this strategy suffers from an intrinsically low throughput, variability and difficulty in accessing relevant control tissue (Eugène et al., 2014; Bak et al., 2024).

Three-dimensional brain organoids, which mimic the early stages of human neurodevelopment, offer a powerful tool for studying some aspects of brain function and the mechanisms underlying disorders that affect brain growth or organization (Lancaster et al., 2013; Amin and Paşca, 2018; Birtele et al., 2025). Although more complex than other *in vitro* systems, cerebral organoids can recapitulate some features of neural network activity, exhibiting distinct oscillations due to excitatory/inhibitory imbalance (Trujillo et al., 2019). Recent advances in organoid technology have enabled the generation of cerebral dorso-ventral assembloids, fused structures combining cerebral cortex and ganglionic eminence organoids. These assembloids facilitate the integration and functional connectivity of both excitatory and inhibitory neurons, allowing more accurate modeling of neural circuits (Birey et al., 2017).

This review summarizes the current knowledge on the use of human brain organoids to model epilepsy, with a particular focus on organoids harboring FCD-linked mutations.

## 2 Focal cortical dysplasia

The latest reported data estimates the prevalence of malformations of cortical development at almost 20% (Blumcke et al., 2017). Among them, FCD was the most common type, particularly predominant in children (around 50% of malformations of cortical development were reported in pediatric cases) (Blumcke et al., 2017). Seizures onset can occur during the neonatal period, early childhood (61% before age 5) and adolescence (93% before age 16), although epilepsy may also occur in adulthood (Cohen et al., 2022).

In 2011, the International League Against Epilepsy (ILAE) introduced the now widely recognized three-levels clinicopathological classification (Blümcke et al., 2011). Specifically, FCD type I was defined as a lesion with architectural disorganization of the cortical layers and cellular abnormalities such as small immature neurons, hypertrophic pyramidal neurons, and neurons with disoriented dendrites (Blümcke et al., 2011). FCD Type II, in addition to abnormal cortical lamination, is characterized by dysmorphic neurons, and is further subdivided into type IIa and type IIb due to the absence or presence of balloon cells, respectively (Blümcke et al., 2011). Both dysmorphic neurons and balloon cells exhibit protein profiles similar to pyramidal neurons but lack those typical of interneurons (Lamparello et al., 2007). This specific protein profile suggests that their progenitors come from the ventricular zone rather than the medial ganglionic eminence (Lamparello et al., 2007). Additionally, balloon cells express neuronal and glial proteins, along with doublecortin, a marker of immature neurons, suggesting that they maintain an embryonic-like state (Lamparello et al., 2007). FCD type II also exhibits excessive cellular cortical gliosis, proliferation and hypertrophy of astrocytes, and microglial activation (Boer et al., 2006; Sisodiya et al., 2009). Microglial cells accumulate around dysplastic neurons and blood vessels, contributing to a neuroinflammatory environment (Boer et al., 2006). Furthermore, their involvement in aberrant synaptic remodeling mechanisms, including loss of dendritic spine density, has been reported potentially through the activation of the complement system (Rossini et al., 2023).

Finally, FCD type III, is a form of cortical dysplasia which usually leads to an abnormal cortical lamination, associated with other main brain lesions, such as hippocampal sclerosis (IIIa), tumors (IIIb), vascular malformations (IIIc), or early damage lesions (IIId), whose symptoms depend on the dominant lesion (Barkovich et al., 2005).

Despite numerous correlation analyses between focal lesion location and age of onset of epilepsy in FCDs, conflicting and variable data have been reported. However, it would appear that FCD I is commonly located in the temporal lobe, while FCD II in the frontal lobe and frontocentral area (Palmini et al., 2004).

Subsequent advances in imaging and molecular genetics led to a formal update of this classification in 2022, introducing integrated, multilayered diagnostic criteria and new FCD subcategories such as mild malformations of cortical development (mMCDs) and cortical malformation with oligodendroglial hyperplasia (MOGHE) (Najm et al., 2022).

As widely described in the following paragraph, a dysfunction in gamma-aminobutyric acid (GABA)ergic transmission is involved in FCDs pathogenesis, with an imbalance of the excitation/inhibition (E/I) ratio shifted toward excitation with consequent neuronal hyperexcitability (Talos et al., 2012; Abdijadid et al., 2015; Blauwblomme et al., 2019; Banerjee et al., 2020). Despite the recent identification of somatic mutations in FCD Type I and II (Macdonald-Laurs et al., 2025), the pathophysiology of FCDs remains incompletely understood. Above all, it remains unclear whether functional network defects are due to early, abnormal cellular differentiation of cortical neuronal subtypes rather than their late, abnormal functional maturation. Up to now there is not an approved target medical treatment. The surgical resection is the best treatment option with up to 70% seizure free after surgery in FCD II and 50–60% seizure free in FCD I (Lamberink 2021). Several factors, including epigenetic dysregulation, cortical developmental protein alterations, inflammatory processes and genetic mutations, could contribute to the development of the FCDs (Winden et al., 2015; Fang et al., 2025). Regarding FCD type II, there is strong evidence supporting the involvement of somatic gain-of-function mutations in genes within the mammalian target of rapamycin (PI3K–AKT–mTOR) signaling pathway, which is crucial for controlling cell growth, neuroglial proliferation, and homeostasis during cortical development (Mirzaa et al., 2016; Pirozzi et al., 2022; Krochmalnek et al., 2023). When mTOR-activating mutations occur, they result in constitutive hyperactivation of the mTORC1 complex, leading to increased S6 phosphorylation, cellular hypertrophy, laminar disorganization, and distinctive cytological alterations (Mirzaa et al., 2016; D'Gama et al., 2017). Further investigations have identified somatic loss-of-function mutations in TSC1/TSC2, genes commonly linked to tuberous sclerosis (D'Gama et al., 2017; Lim et al., 2017). FCD II cases have also revealed somatic or germline, along with very rare “two-hit” germline and somatic, loss-of-function mutations in DEPDC5, NPRL2 and NPRL3, components of the GATOR1 complex, which negatively regulates mTORC1 (Ricos et al., 2016; Weckhuysen et al., 2016; D'Gama et al., 2017). These genetic alterations manifest in a highly comparable histopathological and molecular phenotype, converging on mTOR pathway dysregulation (Weckhuysen et al., 2016; D'Gama et al., 2017). Interestingly, mTOR mutations were observed in just a small percentage of lesion cells in FCD human surgical tissues and animal models, rather than in the whole sample, supporting a somatic mosaic instead of a homogeneous phenotype (D'Gama et al., 2017; Sim et al., 2019; Zhang et al., 2020; Kim et al., 2024). The allele frequency of these mutations has been shown to be extremely low, frequently below 1%, but nevertheless sufficient to support a clinically relevant epileptic phenotype and to provoke spontaneous seizures and abnormal network electrophysiological activity (D'Gama et al., 2017; Sim et al., 2019; Zhang et al., 2020; Kim et al., 2024). This suggests that a remarkably low “critical threshold” of mutated cells is enough to initiate diffuse hyperexcitability. In contrast, FCD type I exhibits a more heterogeneous, though less clearly defined, genetic background. Notably, somatic mutations in SLC35A2 have been linked to FCD type I and mMCD (Winawer et al., 2018).

## 3 Mechanisms underlying Excitatory/Inhibitory imbalance in FCDs

Functional circuits in the neocortex are based on the precise organization and interaction of excitatory and inhibitory neurons. Thus, a central question in cortical development is how excitatory and inhibitory interneurons integrate with each other to reach their final connectivity in the neocortex.

Medial Ganglionic Eminence (MGE)-derived interneurons distribute through the neocortex following the inside-out pattern (Valcanis and Tan, 2003; Hammond et al., 2006), but they invade their corresponding layer only after the glutamatergic projection neurons have settled (Pla et al., 2006; Miyoshi and Fishell, 2011). In fact, the disruption of the normal layering of glutamatergic projection neurons affects and induces changes in the migration of MGE-derived interneurons (Lodato et al., 2011). Thus, these studies suggest that the arrangement of projection neurons significantly affects the migration of MGE-derived interneurons, probably due to the production of layer-specific signals (Bartolini et al., 2013).

Brain function depends on a finely regulated Excitatory/Inhibitory (E/I) balance, which exerts a critical role in controlling spike rate and information processing. E/I balance requires precise connections through dynamic processes involving neurotransmitter receptors, transporters, scaffolding proteins, and cytoskeleton. Perturbation of this balance is a hallmark of many neurological disorders (Ghatak et al., 2021).

Intercommunication between excitatory and inhibitory neurons is the key point of a functional neural network and the balance between these two forces is critical for normal cognition and memory (Nelson and Valakh, 2015), as they control firing patterns of neurons in mice (Crocco et al., 2025). Changes in this E/I homeostatically regulated pattern could destabilize the network activity and induce compensatory mechanisms, such as modulation of postsynaptic strength and neurotransmitter release probability, restoring the default circuit characteristics (Sukenik et al., 2021). Indeed, E/I balance usually refers to a stable global level of activity within a particular circuit, even though individual groups of neurons may exhibit transient imbalances and may be dynamic over time (Sohal and Rubenstein, 2019).

Consequently, what are the functional implications of a disrupted E/I balance? E/I balance is delicately maintained in mature neurons and microcircuits by a variety of homeostatic mechanisms, to regulate overall neural circuit function. Any perturbation in the E/I ratio in the gain of either excitation or inhibition, regardless of the specific mechanism, could contribute to aberrant network activity and hence to neural diseases. The E/I imbalance hypothesis has been postulated to underlie brain dysfunction across neurodevelopmental, neuropsychiatric, and neurodegenerative disorders, including epilepsy, autism spectrum disorder (ASD) or schizophrenia (Foss-Feig et al., 2017).

FCDs are characterized by seizures development, caused by hyperexcitability consequent to alterations of the E/I balance (Talos et al., 2012; Abdijadid et al., 2015; Blauwblomme et al., 2019; Banerjee et al., 2020). The traditional view assumes that the etiology of epilepsy is driven either by reduced inhibitory synaptic transmission, by an increased excitatory one, or a combination of both mechanisms. However, increasing evidence suggests that this view is oversimplistic. Recent findings indicate that increased inhibition (inhibition being defined as bona fide inhibitory synaptic input) can similarly contribute to epileptogenesis, specifically through the action of GABA acting as a depolarizing—rather than hyperpolarizing—neurotransmitter in certain pathological contexts or developmental stages (Blauwblomme et al., 2019; Banerjee et al., 2020).

### 3.1 Chloride homeostasis and developmental regulation of GABA signaling

In mature neurons, classical inhibitory signals are generated by an inwardly directed flux of chloride (Cl^−^), through Cl^−^-dependent GABA_A_ receptor channels opened by GABA. This leads to cellular hyperpolarization with a reduced probability of action potential generation. Intracellular chloride homeostasis is regulated primarily by two cation Cl^−^ cotransporters encoded by the SLC12 family genes: the neuron-specific K^+^-Cl^−^ cotransporter 2 (KCC2) and Na^+^-K^+^-2Cl^−^ cotransporter 1 (NKCC1) (Hebert et al., 2004). KCC2 maintains a low intracellular Cl^−^ concentration through extrusion of Cl^−^ by harnessing K^+^ gradients (Lu et al., 1999), while NKCC1 utilizes the inward Na^+^ and K^+^ gradient to drive Cl^−^ influx (Yamada et al., 2004). NKCC1 is also present in glial cells and emerging evidence indicates its up-regulated expression in response to inflammatory stimuli in neurodevelopmental and neurological disorders (Luo et al., 2014; Tóth et al., 2022).

Overall, the relative expression and activity of NKCC1 and KCC2 determine the value of the Cl^−^ equilibrium potential and, consequently, the polarity of GABAergic signaling (Ben-Ari et al., 2012). In mature healthy neurons, the intracellular Cl^−^ concentration is low, thus the activation of the GABA_A_ receptor will cause a Cl^−^ influx and hyperpolarization of the membrane potential, reducing the probability of action potential initiation. On the other hand, during early development, when the internal Cl^−^ concentration is higher, due to the delayed expression of the Cl^−^ exporter KCC2, GABA_A_ receptor activation results in an outwardly directed flux of Cl^−^ and neuronal depolarization (Rivera et al., 1999). As development proceeds, increased KCC2 expression lowers intracellular chloride levels, shifting GABAergic signaling from the depolarizing to the hyperpolarizing direction (Van van Hugte et al., 2023).

Inhibitory inputs are fundamental for regulating the E/I balance and a mismatch between the GABAergic and glutamatergic synaptic drive could either result in the prevention of synapse formation and development or cause excitotoxicity. The switch of GABA action from the depolarizing to the hyperpolarizing direction during brain development is critical for normal cortical maturation, as depolarizing GABA regulates cell proliferation, cell migration and synaptogenesis (Ben-Ari, 2002; Kasyanov et al., 2004; Peerboom and Wierenga, 2021). Alterations in depolarizing GABA may impair synapse formation and circuit stability, leading to seizure generation and altered neurodevelopment in epilepsy, including FCDs (Moore et al., 2017; Bozzi et al., 2018; Pisella et al., 2019).

Most recent studies support the current paradigm that disrupted KCC2 activity leads to seizures by enhancing intracellular Cl^−^, thus promoting depolarizing GABAergic signaling, and consequently an E/I imbalance in selected brain regions (Van van Hugte et al., 2023). It has been observed that KCC2 might be one of the principal contributors behind epileptogenesis, both disrupting network activity and conferring vulnerability to disinhibition through activity-dependent mechanisms, resulting in a lower seizure threshold (Liu et al., 2020). Not surprisingly, several groups confirmed GABA_A_ receptor-mediated depolarization and a functional implication of NKCC1 and KCC2 in FCD patients, which is attributed to their altered expression and localization (D'Antuono et al., 2004; Talos et al., 2012; Ben-Ari, 2017; Blauwblomme et al., 2019; Liu et al., 2022). These results have paved the way for further research into NKCC1/KCC2 as new potential therapeutic targets for FCDs.

### 3.2 Beyond chloride dysregulation: additional mechanisms contributing to E/I imbalance

Beyond its role in regulating chloride homeostasis, KCC2 also plays a crucial role in functional maturation, interneuron migration, dendritic spine organization, and AMPA receptor trafficking, highlighting its influence on both inhibitory and excitatory synaptic function (Llano et al., 2015; Peerboom and Wierenga, 2021). A sudden disruption of KCC2 activity may contribute to activity-dependent disinhibition, potentially explaining the rapid onset of seizures (Lee et al., 2011). Moore et al. observed that brain-derived neurotrophic factor (BDNF) is an important negative regulator of KCC2 expression (Moore et al., 2017). Since BDNF levels increase during seizure activity, they hypothesized that both glutamate- and BDNF pathways converge to decrease KCC2 activity, conferring susceptibility to seizures (Moore et al., 2017).

Nevertheless, while KCC2 dysfunction and depolarizing GABAergic signaling are highly relevant in E/I imbalance, they are not the only factors responsible for the alteration of the E/I balance in epilepsy and neurodevelopment. An E/I dysregulation might cause epileptogenesis not only in patients with genetic or acquired epilepsies, but also in patients with neurodevelopmental disorders with epilepsy as a comorbidity, suggesting common underlying mechanisms.

In this context, molecular defects in synaptic structure and function, involving crucial scaffolding proteins like neuroligins and neurexins, may act as common triggers for epilepsy (Bozzi et al., 2018) as their disruption compromises synaptic alignment and directly impairs the organization of a balanced neuronal signaling (Canitano and Bozzi, 2023).

In addition to alteration in chloride homeostasis and GABA polarity, a reduced number or dysfunction of parvalbumin-positive (PV^+^) inhibitory interneurons can lead to increased susceptibility to seizures, given their critical role in regulating the synchronization of neuronal firing necessary for the maintenance of the E/I balance (Leitch, 2024). Alterations in the number, morphology and function of PV^+^ interneurons have been reported in various forms of epilepsy and accumulating evidence indicates that these alterations are particularly prominent in FCDs (Juarez and Martínez Cerdeño, 2022).

Multiple studies demonstrate significant alterations in PV^+^ interneurons across FCD subtypes. Reduced PV^+^ interneuron density, most pronounced in FCD type IIb, and a decreased ratio of PV^+^ cells relative to the total interneuron population across cortical layers have been reported (Liang et al., 2020). Consistently, scattered and markedly reduced numbers of PV^+^ neurons have been observed in dysplastic cortical regions (Thom et al., 2003). Together, these findings suggest that impaired PV^+^-mediated inhibition is a common pathological feature contributing to cortical hyperexcitability in FCDs. Inhibitory synaptic drive is a fundamental component of neural networks, and our understanding of its role in network dynamics and function is constantly evolving. However, as briefly discussed, various other molecular and cellular mechanisms also contribute to the altered E/I balance observed in epilepsies, with the depolarizing and immature action of GABA emerging as a key factor among possible contributing mechanisms. Thus, depolarizing and immature GABA signaling, and mechanisms leading to it, represents a promising area to explore as an additional therapeutic target for FCD. Finally, because seizures are not only a debilitating symptom but also contribute to further neurological decline, additional investigations are well worth the effort.

## 4 Models for the study of FCDs

**Figure 1:**
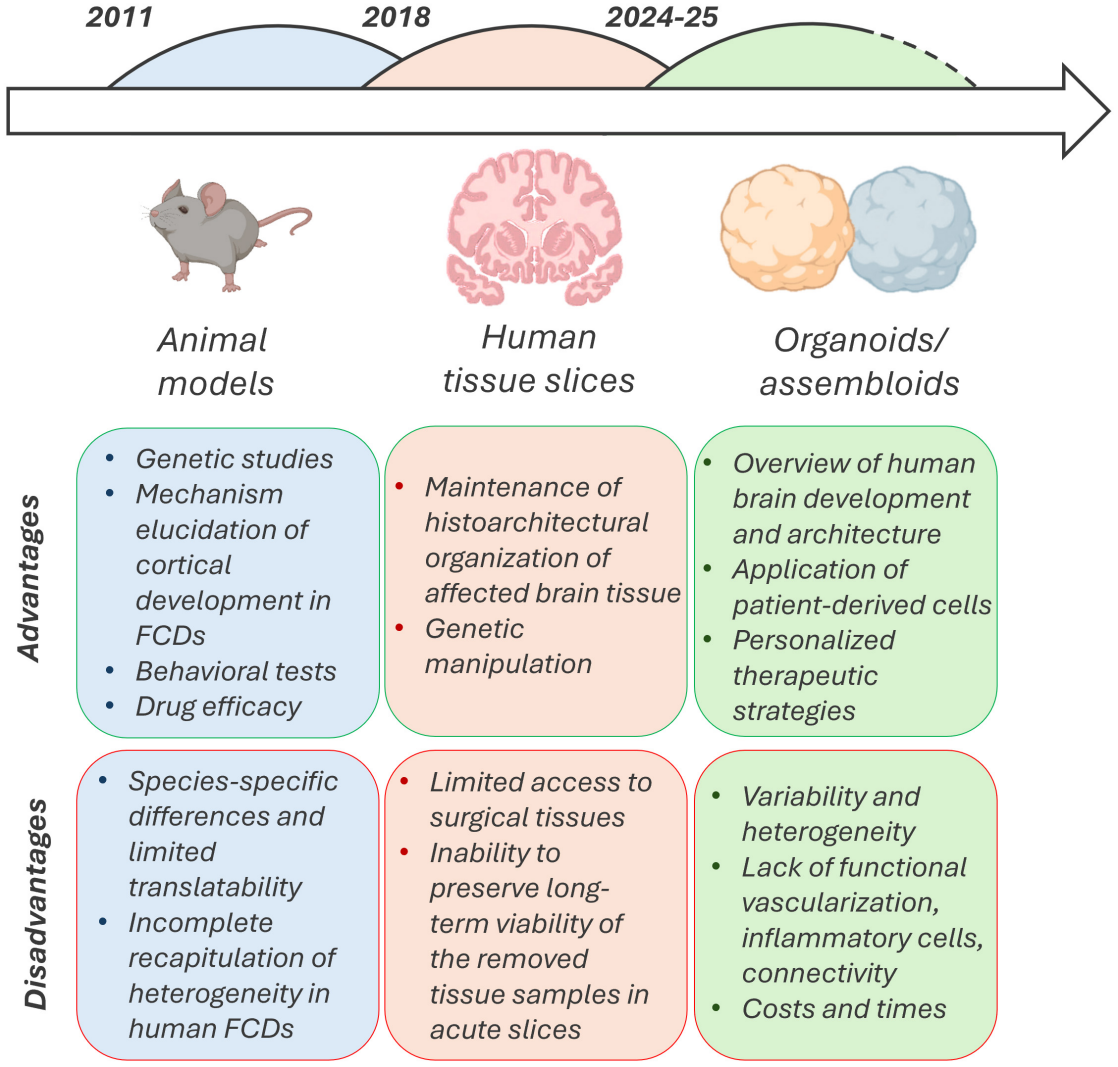
Current models for studying focal cortical dysplasias (FCDs) with their advantages and limitations. Although there are differences between species and they do not fully replicate the heterogeneity of human FCDs, animal models are essential for understanding the molecular and genetic mechanisms underlying cortical development in FCDs and for testing the efficacy of drugs. On the other hand, human tissues retain brain organization and are easy to genetically manipulate, but limited access to surgical tissues due to ethical issues and the inability to preserve long-term viability in harvested samples make them difficult to use as comprehensive models of FCD. 3D cell models (organoids and assembloids) are a fundamental platform for gaining an overview of human brain development and, as they are usually formed from human induced pluripotent stem cells (hiPSCs) derived from patients, they allow for the easy development of personalized and specific therapeutic strategies. However, organoids are known for their high costs, variability and heterogeneity between samples and, in order to achieve complete network histology, it is necessary to add different types of inflammatory cells, astrocytes and oligodendrocytes to the cultures. The figure was formed with Biorender open source software.

With the progression of new technologies, methods for investigating FCDs are also constantly evolving. Since FCDs arise from complex developmental and genetic mechanisms, no single experimental model can fully recapitulate their pathology. Hence, in view of a personalized type of medicine, a combination of animal and human-based models has been employed, allowing the study of FCDs across different biological scales, from cortical development to network dysfunction and epileptogenesis (Figure 1).

### 4.1 Animal models of FCDs

Animal models are crucial for understanding the mechanisms underlying many neurodevelopmental disorders, including FCDs. Several approaches have been employed to create murine models capable of reproducing features of FCDs, specifically disorganized cortical architectures and neuronal abnormalities and impaired function. Among them, the most widely used are chemically induced models; prenatal exposure to methylazoxymethanol acetate interferes with neuronal proliferation by producing laminar migration and disorganization which mimic human FCDs abnormalities (Colciaghi et al., 2011, 2014). More recently, *in utero* electroporation techniques have been used to introduce constructs or mutations of genes implicated in cortical development, recreating lesions that faithfully mimic FCDs. Other approaches include transgenic knock-in mice harboring mTOR mutations individually associated with forms of human FCD such as TSC1 and TSC2 (Feliciano et al., 2011; Liang et al., 2020), mTOR itself (Lim et al., 2015), Rheb (Hsieh et al., 2016), and DEPDC5 (Marsan et al., 2016; Ribierre et al., 2018). In human FCD, often the mutations are somatic rather than inherited. Therefore, the described transgenic models in which mutations are introduced in the germline can reproduce certain features, such as neuronal dysmorphism, the presence of balloon cells, the disruption of E/I balance and local connectivity, but they do not recapitulate the complex mosaicism of human FCD.

### 4.2 Human tissues and organotypic cultures

Many biological functions are well conserved throughout evolution, making animal models valuable for uncovering mechanisms of epileptogenesis and assessing the efficacy of pro- or anti-convulsant drugs. However, the human brain exhibits distinct characteristics that set it apart in its organization and complexity, limiting the translational relevance of such models. Difficulties in translating animal data to humans particularly concern epileptic disorders which do not respond to drugs. Due to different pharmacokinetics, dynamics and unexpected side effects of drugs in humans, therapeutic molecules tested on animal models often poorly translate to clinics. An emerging alternative to overcome these limitations is the use of human brain tissue obtained during surgery or immediately postmortem.

These acute slices maintain their viability *in vitro* for a duration of a few hours, limiting extended or iterative experimental procedures (Bak et al., 2024). However, these slices enable immediate examination of human neuronal physiology after excision and offer insights into human synaptic function in pathological conditions (Eguchi et al., 2020).

Baldassari et al. (2025) recently investigated the cellular identity and functional impact of somatic mutations underlying FCD Type II, using single-nucleus resolution techniques on cortical tissues obtained surgically from patients with genetically mosaic FCDII. They specifically noticed that dysregulated gene expression related to synapses, neuronal development, and mitochondrial metabolism was present in both mutated and unmutated cells within affected tissue, suggesting that both “cell-autonomous” and “non-cell-autonomous” mechanisms might contribute to FCDII-associated epilepsy (Baldassari et al., 2025). However, in addition to the inability to preserve long-term viability of the removed tissue samples, the main challenge of this approach is the limited accessibility of surgical tissue. Consequently, recent studies have increasingly focused on organotypic slice cultures obtained from human tissues, which could be kept alive for several weeks (Schwarz et al., 2017; Wickham et al., 2018; Brown et al., 2024). These cultures provide significant advantages: they effectively preserve the original histoarchitectural organization of affected brain tissue, including layered organization, local synaptic connectivity, and pathology-related cellular alterations, while maintaining the functional integrity of the tissues (Bak et al., 2024). This approach allows longitudinal studies of neuronal network properties and enables viral-mediated delivery of protein of interest for functional investigation. Importantly, organotypic slices can sustain epileptic activity *in vitro* for 3 or 4 weeks, thereby enabling the observation of epileptic phenomena's progression and the assessment of long-term and chronic effects of potential therapeutic agents (Schwarz et al., 2017; Wickham et al., 2018; Brown et al., 2024). Furthermore, these cultures allow for genetic manipulation through viral transfection to directly study the functional impact and pathophysiological mechanisms of defined mutations (Schwarz et al., 2017; Wickham et al., 2018; Brown et al., 2024). Thus, organotypic brain slices offer a model that more closely mimics an *in vivo* environment than primary single-cell cultures, partly by preserving their complex three-dimensional structure. However, despite maintaining cellular connections, these cultures face significant limitations due to axotomy, which may lead to the loss of target innervation and, consequently, neuronal cell death (Humpel, 2015).

### 4.3 3D cellular models

Due to challenges in accessing to primary human brain tissues, among these years, pluripotent stem cells (PSC) have been identified as a very promising tool for neurobiology, allowing the *in vitro* recapitulation of many developmental events that occur during brain organogenesis and/or differentiation of specific cell types (Gaspard and Vanderhaeghen, 2010).

During the past two decades, cellular studies have been conducted to establish the best reliable *in vitro* model to study neurodevelopmental mechanisms and disorders. Various protocols and analysis have been matured for neural differentiation into many regions of the cortex: striatal projection neurons (Delli Carri et al., 2013; Arber et al., 2015; Fjodorova et al., 2015), isocortex (Gaspard et al., 2008; Bertacchi et al., 2015; Pandolfini et al., 2016), spinal cord (Wichterle et al., 2002), general lateral ganglionic eminence (LGE)- and MGE-cell type culture (Noakes et al., 2019), hypothalamus (Miwata et al., 2023), and hippocampus (Sarkar et al., 2018; Dunville et al., 2022).

Stem cell-derived neurons are also able to mature functionally, expressing voltage gated cation channels quite similar to their respective *in vivo* wild-type (Barth et al., 2014).

While stem cell-derived neurons have proven invaluable in two-dimensional (2D) culture systems, it has been suggested that accurately modeling the intricate architecture of the developing brain requires three-dimensional (3D) growth platforms. Indeed, 3D models, such as spheroids and organoids, have provided new highlights into questions regarding development (Birey et al., 2017; Chiaradia and Lancaster, 2020; Sidhaye and Knoblich, 2021; Chiaradia et al., 2023), molecular interactions (Bertacchi et al., 2024) and pathological modeling (Iovino et al., 2015).

3D modeling brain organogenesis *in vitro* has continued to advance, first with the optimization of protocols for the neuralization of floating Embryoid bodies (EBs) in serum-free medium, known as SFEBq (serum-free floating culture of EB-like aggregates) (Watanabe et al., 2005; Eiraku et al., 2008), then with cerebral organoids (Lancaster et al., 2013). In cerebral organoids, neural fate was shown to be acquired on the surface of aggregates, and these neural tube-like units seemed different from neural rosettes because of their larger lumen and an epithelium with a self-determined dorsoventral-rostro caudal axial polarity (Chiaradia and Lancaster, 2020). The breakthrough came with the introduction of extracellular matrix components in the form of Matrigel: dissolved Matrigel can, in fact, lead to the production of a fully 3D self-organizing neural tissue *in vitro*. The combined effect of the gel's physical attributes, such as rigidity, alongside the crucial signaling cues provided by the basement membrane ligands in Matrigel, supports organoid genesis, acting as both a scaffold and a modulator of biological functions, including tissue polarity and cell migration. The timed addition of Matrigel into 3D brain organoid protocols strongly promotes the rapid formation of polarized, neural tube-like buds from the neuroepithelial tissue (Benito-Kwiecinski and Lancaster, 2020). Moreover, optimized media transitions and agitation of the tissues can induce the formation of regionalized cerebral organoids with various brain region identities (Paşca et al., 2015) and progenitor zones, including also a subventricular zone (SVZ) with outer radial glia (oRGs) (Lancaster et al., 2013). The resulting neurons subsequently arrange in an “inside-out layering,” where early-born neurons reside in deeper layers, and later-born neurons migrate to more superficial positions (Benito-Kwiecinski and Lancaster, 2020).

Recent single-cell analyses and the development of improved culture conditions have also led to the generation of human cortical organoids with remarkable fidelity to the human brain, including the formation of ordered cortical layers and the recapitulation of cellular diversity and regionalization (Velasco et al., 2019). Lately, it was also proved that organoids could be generated from stem cells from many other species, including non-human primates, thereby establishing a comparative experimental platform to investigate features of human brain specialization (Pollen et al., 2019; Benito-Kwiecinski et al., 2021). However, the formation of a well layered cortical structure is often impaired in cortical organoids. Traditional spherical brain organoids suffer from interior hypoxia and cell death due to limited nutrient diffusion, which eventually triggers the depletion of neural progenitor cells and structural disorganization. The sliced neocortical organoid (SNO) system overcomes these limitations by exposing the interior tissue to the culture environment, thereby preventing necrotic core formation and sustaining neurogenesis over prolonged periods. This advanced methodology allows for the consistent formation of an expanded cortical plate with well-separated upper and deep layers, effectively recapitulating the complex laminar architecture characteristic of the late-stage human embryonic neocortex. SNOs were employed to demonstrate that a genetic mutation in DISC1 associated with psychiatric disorders generates a loss of laminar specificity that is restored upon genetic correction of the mutation (Qian et al., 2020). Therefore, SNOs appear to be the best candidates for studying cortical stratification disorders such as FCD associated with distinct gene mutations.

Beyond structural maturation, another crucial point is the lack of specific microenvironment and sensory inputs that exist *in vivo* during the maturation of brain organoids, limiting their utility in modeling complex and behaviorally defined neurological disorders (Wang et al., 2023). Transplantation of human neurons and brain organoids into the rodent cortex has been defined as an emerging strategy for achieving advanced neuronal maturation and functional integration (Linaro et al., 2019; Xiong et al., 2021; Revah et al., 2022). Many studies demonstrated that grafted brain organoids developed a higher degree of vascularization (Daviaud et al., 2018) and showed progressive transition from the progenitor phases to neuronal and glial maturation *in vivo*, revealing spontaneous rhythmic calcium transients and network activity (Mansour et al., 2018). Recent studies have revealed that cerebral organoids can recapitulate complex inter-regional interactions in a manner like *in vivo*. Specifically, GABAergic interneurons originating from ventral forebrain, were frequently detected in regionalized dorsal forebrain organoids, even in the absence of a ventral component, suggesting an intrinsic potential of the dorsal lineage to generate inhibitory cell (Velasco et al., 2019). This suggests a ventral-dorsal migration similar to the developing forebrain where inhibitory interneurons integrate and connect into cortical circuits (Lancaster et al., 2013). The emergence of these inhibitory populations could be modulated by specific signaling pathways. For instance, Andrews et al. demonstrated that forebrain organoids treated with LIF (Leukemia Inhibitory Factor) promoted an increased proliferation of outer radial glial cells and a subsequent formation of inhibitory interneurons that molecularly resemble those originating from the caudal ganglionic eminence (CGE) (Andrews et al., 2023). Similarly, FGF8 has been identified as a crucial factor in regional identity in brain organoid: by modulating early target genes, there is a strong emergence of inhibitory populations that integrate into the neural circuits (Bertacchi et al., 2024). However, interneurons generated within dorsal forebrain organoids do not accurately reflect the dominant cortical IN subtypes observed *in vivo*. Indeed, distinct interneuron subtypes, including PV^+^, somatostatin (SOM^+^), and CGE-derived populations such as vasoactive intestinal polypeptide (VIP^+^) interneurons, have unique function in cortical microcircuit, mediating perisomatic inhibition, dendritic inhibition, and disinhibitory control, respectively. The presence of CGE-derived interneurons (the VIP^+^ cells) may therefore introduce disinhibitory motifs and interneuron–interneuron (i.e., VIP^+^-SOM^+^) interactions, shaping baseline network excitability, oscillatory behavior, and shunting inhibition. Consequently, unintended variety of IN subtype within organoids may significantly influence E/I balance, a central mechanism in FCD pathophysiology. These considerations underscore the necessity of carefully characterizing interneuron composition when employing organoid models to investigate mechanisms of epilepsy at circuit level.

Thus, recent studies created fused region-specific dorsal and ventral forebrain organoids, known as assembloids, to study the saltatory migration of interneurons from the ventral into the dorsal component (Bagley et al., 2017; Birey et al., 2017; Walsh et al., 2025). Importantly, these migrated interneurons were shown to alter their morphology, establish synapses with dorsal glutamatergic neurons, and form electrically active, functional cortical microcircuits (Birey et al., 2017). One limitation of this approach is the inability to regulate the proportion of GABAergic migrated interneurons. This proportion is essential for the maturation of appropriate cortical network activity, and recent studies have shown that saltatory migration itself is dispensable for their functional integration into the cortical circuit (Crocco et al., 2025). These observations suggest that, in the future, the generation of organoids or SNOs obtained by mixing controlled proportions of dorsal and ventral early neural progenitors could better contribute to modeling functionally optimized cortical networks. Functional analysis remains a definitive benchmark for assessing the maturity and synaptic connectivity of neural circuitry and for the now emerging field of brain organoids. Recordings of neural activity are also instrumental for the development of human electrophysiological data-driven models of epilepsies and other neurodevelopmental diseases. Although a wide range of established electrophysiological modalities has been successfully adapted to 3D cultures, their applications to organoid architecture require a careful balance of technical compromises (Passaro and Stice, 2021).

The first and most commonly used method for measuring neuronal activity and maturity is the patch-clamp electrophysiology, which enables the analysis of passive electrical properties of different types of neurons, the synaptic connectivity between paired neurons and the functional expression of ion channels in slice cultures (Birey et al., 2017). In these years, many studies have also provided detailed high temporal resolution-analysis of specific neurons in brain organoids (Di Lullo and Kriegstein, 2017; Cakir et al., 2019; Trujillo et al., 2019). The accuracy of patch-clamp recordings remains fundamental although in organoid research this technique is not widely used. Indeed, functional analyses in organoids have largely focused on the assessment of network-level activity, characterized by synchronized firing and regional interactions, using calcium imaging and multi-electrode array (MEA) recordings. Calcium imaging has emerged as a powerful technique for live cell monitoring of neural activity in small groups of neurons. Among other examples (Andersen et al., 2020; Sebastian et al., 2023; Kim et al., 2025b), calcium imaging has been used to assess the functional connectivity in cortico-striatal assembloids (Miura et al., 2020), to investigate neuronal behavior in LIF-treated organoid in neurogenic studies (Walsh et al., 2024) or to identify epileptiform-like activity in human brain organoids (Samarasinghe et al., 2021). In addition to being among the few to employ patch-clamp recordings to assess the functional maturation of forebrain organoids, Qian et al. also investigated the developmental GABAergic depolarization switch by monitoring Ca^2+^ transients in response to GABA application. Their findings revealed a high expression of NKCC1 at both days 56 and 84 of differentiation, whereas KCC2 expression was strongly increased at day 84 but remained low at day 56. Quantitative analysis further showed an increased percentage of neurons that did not exhibit a GABA-induced Ca^2+^ rise within the glutamate-responsive population, consistent with a progressive shift toward hyperpolarizing GABA response. These results demonstrate that forebrain organoids successfully recapitulate functional features of neuronal maturation found *in vivo* (Qian et al., 2016).

In addition to being among the first to employ patch-clamp recordings to assess the functional maturation of forebrain organoids, Qian et al. also investigated the developmental GABAergic depolarization switch by monitoring Ca^2^? transients in response to GABA application. Their findings revealed high NKCC1 expression at both day 56 and day 84 of differentiation, whereas KCC2 expression was markedly increased at day 84 but remained low at day 56. Quantitative analysis further demonstrated an increased proportion of neurons that did not exhibit a GABA-induced Ca^2^? rise within the glutamate-responsive population, consistent with a progressive shift toward hyperpolarizing GABA responses. Collectively, these results indicate that forebrain organoids recapitulate key functional features of neuronal maturation observed in vivo (Qian et al., 2016).

Beyond the calcium imaging, recent advances in extracellular recordings and MEAs have enabled long-term, non-invasive, large-scale electrophysiological studies (Kanton and Paşca, 2022). These techniques can be adopted for screening applications (Shafer et al., 2019) and for the analysis of the functional connectivity in both control and disease-model organoids, such as schizophrenia (Walsh et al., 2025) or epilepsy (Yokoi et al., 2021).

### 4.4 Organoids in FCDs

As previously described, 3D modeling of human cortical circuits was made possible by the culture of hiPSCs-derived spheroids and organoids. Avansini et al. (2022) successfully generated human cortical organoids from iPSCs derived from patients with pathologically confirmed FCD Type II. The organoid model accurately reproduced several core hallmarks of this pathology, including impaired cell proliferation, and the presence of characteristic abnormal cells such as dysmorphic neurons and balloon cells (Avansini et al., 2022). The authors characterized 90-day-old organoids using both morphological and immunohistochemical analyses. Notably, only FCD organoids exhibited NeuN? cells with soma approximately twice the size of those observed in control organoids, as well as large, pyramid-shaped MAP2? cells displaying morphological abnormalities consistent with dysmorphic neurons. Furthermore, immunostaining for Nestin and MAP2 revealed several cells with abnormal somatic morphology, including lateral displacement of the nucleus and phenotypic features indicative of both progenitor and neuronal lineages, resembling histologically/morphologically-defined balloon cells (Avansini et al., 2022). Functionally, multi-electrode arrays (MEA) and optogenetic stimulation revealed significant functional defects in these organoids, exhibiting pronounced neuronal network hyperexcitability, characterized by an increase in the number of spikes, mean firing rate, and number of bursts compared to control organoids (Avansini et al., 2022). Moreover, the mutant organoids showed enhanced network connectivity with a higher response rate and increased spread of electrical activity (Avansini et al., 2022). These results show that cellular and functional immaturity impairs the formation of balanced neural networks and makes tissue more susceptible to epileptogenesis, suggesting that these models reproduce not only structural and morphological alterations, but also dynamic network characteristics related to epileptic seizures.

Among the first studies using human organoids to investigate FCD associated with mTOR dysfunction, Blair et al. (2018) developed human cortical spheroid models derived from Tuberous Sclerosis (TS) patients. These organoids reproduced the histopathological features observed in TSC tubers and mTOR-related FCDs, particularly FCD type II, demonstrating that biallelic loss of TSC2 in human cortical progenitors is sufficient to generate dysmorphic cells, glial augmentation, and tissue disorganization (Blair et al., 2018). Recently, Lu et al. investigated the effects of the NPRL3 mutation on neuronal development and mTOR activation, using cortical organoids derived from FCD II patients (Lu et al., 2024). The authors reported that the patient's cortical organoids were larger than controls and showed increased pS6?, a marker of mTOR pathway activation, and enhanced number of NeuN? neurons, confirming that this model is able to reproduce a cellular phenotype consistent with FCD II (Lu et al., 2024).

An innovative model of FCD II was obtained using brain organoids obtained from patient-derived human astrocytes (Xu et al., 2025). Regionalized Dorsal (DFOs) and ventral (VFOs) forebrain organoids were generated to understand whether the region of origin influences the onset of the pathology. The authors demonstrated that the pathology is strongly dependent on dorsal developmental programs. In fact, patient-derived DFOs showed key features of FCD II, including dysmorphic neurons, balloon cells, and marked astrocytic reactivity, while these alterations have not been reported in VFOs (Xu et al., 2025). Furthermore, some FCD organoids unexpectedly presented cardiomyocyte-like cells, a phenomenon associated with hyperactivation of the BMP and WNT pathways. These cells not only alter tissue organization, but contribute to neuronal hyperactivity, with epileptiform discharges that reproduce the electrical dysfunction of patients (Xu et al., 2025).

Finally, a recent preprint study on bioRxiv used loss-of-function in patient-derived cortical organoids “mosaics” of DEPDC5, the regulatory gene of mTOR (Maletic et al., 2025). The authors showed increased mTOR activity, alterations in neuronal rosette density, neurons with dysmorphic morphology, and hyperexcitability. In addition, transcriptional changes suggest alterations in neuronal differentiation pathways (Notch, Wnt) and in genes associated with synapses and epilepsy, strengthening the idea that organoids with somatic mutations in the mTOR pathway may reproduce FCD phenotypes (Maletic et al., 2025).

A key point in understanding the causes of dysplasia is the contribution of mechanisms that regulate proliferation, differentiation, migration, and maturation of neural subtypes during corticogenesis in neuronal lineages of patients with FCD. This aspect has not yet been fully studied. In the future, fine longitudinal scRNA-seq analysis of FCD cortical organoids or SNOs may help to better address this aspect. Nevertheless, a notable limitation of current FCD organoids mentioned above is the use of gene editing methods, such as CRISPR/Cas9, to uniformly introduce mutations or achieve high percentages of genetic alterations throughout the entire organoid. Although this approach is often adopted for experimental ease, it does not accurately represent the somatic mosaicism characteristic of patients and could therefore affect how results are interpreted.

Since the involvement of microglia in FCD pathologies is such a crucial point in the inflammatory process, current researches are focusing on the prospect to recreate an *in vivo*-like microenvironment, also mixing neuronal and microglial cells (Mordelt et al., 2025; Wendt et al., 2025; Wogram et al., 2025). These studies offer a good strategy and platform to understand the intercommunication and neuro-immune crosstalk that influence the maturation and function of the cerebral network.

In a preliminary work, Mordelt et al. (2025) developed long-term co-maturation of human microglia derived from iPSC, Neurogenin-2 induced glutamatergic neurons and astrocytes. In these co-cultures, microglia acquired the characteristic ramified morphology with an upregulation of typical markers such as P2RY12 and TMEM119. Although it is a 2D model, this is one of the first examples that fully recapitulates the characteristic microglia-neuron interactions observed *in vivo*, with microglia forming direct contacts with dendrites and axon components. Another relevant example was recently reported by Wogram et al. (2025), who developed a valid platform of brain organoids containing hiPSC-derived microglia. Interestingly, microglia properly integrated into organoids, acquiring a morphology and gene profile very similar to that of primary human microglia (Wogram et al., 2025). Furthermore, the model also supports functional characteristics of microglia by responding to pro-inflammatory stimuli (Wogram et al., 2025).

Finally, despite its limited application to FCD to date, brain organoids incorporating microglia represent an advanced and promising model with broad applicability in exploring the mechanisms underlying neurodevelopment and neurodegeneration, evaluating pharmaceutical agents, and advancing regenerative treatments.

## 5 Limitations and conclusions

This review focuses specifically on how brain organoids have been utilized in various studies to elucidate the mechanisms underlying epileptogenesis and neuronal hyperexcitability. Although the number of studies on epilepsy employing brain organoids is relatively small, the results generated thus far demonstrate its great promise as a future cornerstone of epilepsy research. However, despite significant progress, the application of brain organoids in neurodevelopment research, particularly for modeling FCD, continues to be constrained by substantial limitations, preventing them from fully replicating the complexity of the human brain.

First, it is critical to recognize that cerebral organoids do not constitute a faithful baseline model of normal human cortical morphogenesis. Indeed, organoids intrinsically show structural abnormalities that resemble cortical malformations, even when generated from “healthy” control iPSC lines. Hence, some “disease-like” phenotypes observed in patient-derived organoids may reflect intrinsic limitations of the organoid system itself rather than bona fide patient-specific pathology, confounding the interpretation of FCD-associated phenotypes (Krefft et al., 2021). In this context, as widely described in the literature, genetic mosaicism, especially involving genes in the mTOR signaling pathway, represents one of the most accredited pathogenic mechanisms underlying FCD, particularly type II forms. Mosaicism, the complex interaction between mutated and unmutated cells, and the need for advanced neuronal maturation represent methodological and biological limitations that are still not completely overcome by FCD organoid models currently in use. To address these limitations, particularly in the case of somatic mosaicism mutation such as those found in FCD type II, brain-derived cells represent a necessary rather than merely alternative source, as brain-specific mutations would not be captured in fibroblasts. Evidence indicates that human iPSCs can be generated from brain tissue harboring mosaic somatic mutations, providing an essential tool for modeling neurological diseases in which mutations are confined to neural lineages (Hester et al., 2009; Ruiz et al., 2010). Another promising strategy is the controlled introduction of mosaic mutations into human organoids which could help overcome current limitations and make these models an extraordinarily valuable experimental tool, offering a platform more faithful to human neurobiology.

An additional critical issue arises from the variability and heterogeneity among organoids, even when derived from the same stem cell line. Distinct organoids or their derivatives in separate experiments can exhibit different maturation structures and time scales, thus complicating the acquisition of reproducible data. By exploiting new technologies, such as microfluidic platforms or bioreactors, more homogeneous and controllable organoids will hopefully be generated, increasing their reproducibility.

Furthermore, current “neuron-only” *in vitro* models, frequently fail to reach mature brain stages as many essential cell populations, such as oligodendrocytes, vascular cells, and microglial cells, are either absent or under-represented (Nieto-Estévez and Hsieh, 2020). This consequently limits the potential to investigate key and relevant aspects of FCD pathology, such as glia-neuron interactions, myelination, or modulation of the brain microenvironment. Indeed, through single-cell RNA sequencing (scRNA-seq) studies of “cell-to-cell communication” conducted on mTORopathies tissues, including FCDs, it has been shown that the communication networks between neurons, astrocytes, endothelial, and immune cells are altered, exhibiting distinct changes in synaptic signaling, the extracellular matrix, and glia-neuron interplay (Scheper et al., 2025).

Another structural limitation of brain organoids is the lack of functional vascularization that ensures adequate distribution of oxygen and nutrients in the internal regions, leading to the formation of a necrotic core and making long-term maintenance more difficult, worsening the sustainability and viability of the tissue. SNOs have proven useful in limiting anoxic necrosis but do not reproduce the interactions between neural cells and vessels. To address the lack of vascularization and the absence of non-neuronal cells, organoids “vascularized” or organoids supplemented with hiPSCs-derived microglia have been generated (Rizzuti et al., 2024; Han et al., 2025; Wogram et al., 2025).

A key challenge also remains the reconstruction of functional connectivity within organoid systems.

While early models successfully generated multiple brain regions within a single organoid (Lancaster et al., 2013; Quadrato et al., 2017), their anatomical organization was largely stochastic. Thus, to model the contribution of cell types derived from other cortical regions or to model the interactions and migration between different tissues, more complex models are needed. Assembloids mitigate this issue by combining distinct organoids or introducing missing cell types, thereby enabling additional emerging properties of tissue development (Kanton and Paşca, 2022). The strategy of fusing regionalized neural organoids has been widely adopted to study different features of brain function, including interactions between the cortex and other brain regions, such as the thalamus (Xiang et al., 2019) and striatum (Miura et al., 2020), as well as the hypothalamic–pituitary axis (Kasai et al., 2020). This approach is particularly relevant for FCD, in which the epileptogenic zone may extend beyond the primary lesion to subcortical structures, including the thalamus and basal ganglia. Moreover, it provides a valuable platform for modeling the complex inhibitory circuits involved in FCD pathophysiology. Given that certain forms of FCD are linked to the dysfunction of PV^+^ interneurons, model systems should prioritize the reconstruction of the deepest cortical layers, where these neurons mainly innervate and exert their inhibitory control (Medici et al., 2016). Indeed, fusion of regionalized cortical organoids with MGE-derived ones, enable the direct migration and functional integration of PV^+^ interneurons into these specific laminar targets (Birey et al., 2017).

In conclusion, human brain organoids and assembloids will allow addressing an important question in the field, namely the relative contribution of neurodevelopmental defects vs. those arising at later stages of CNS development. Furthermore, to promote the transfer of pathophysiological findings from organoids to the human brain, it is essential to first understand whether these models accurately reproduce the neural circuits observed *in vivo* and to address the ethical issues that arise.

**Figure 2:**
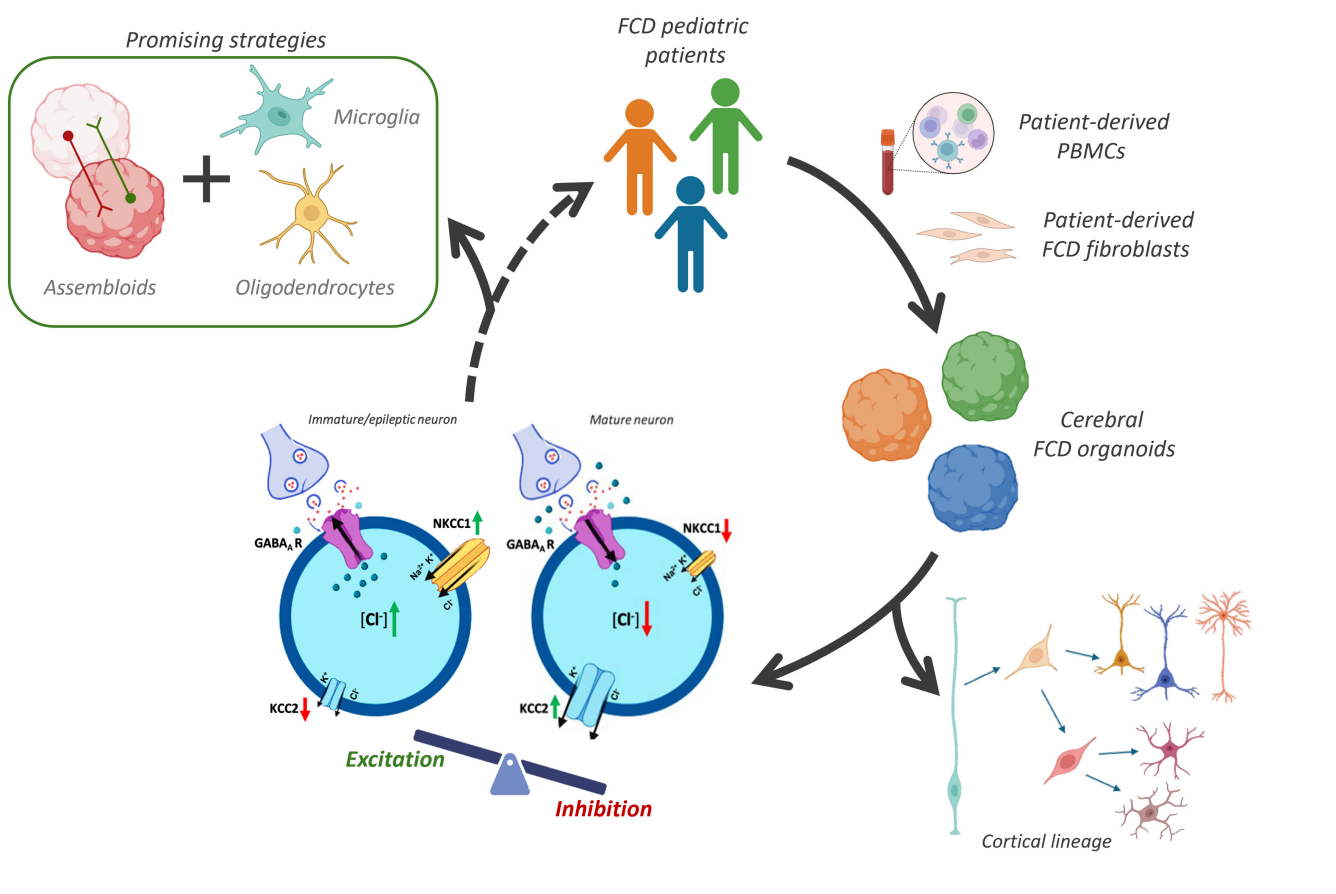
Experimental workflow and future perspectives in focal cortical dysplasia (FCD) modeling. The schematic illustration shows the transition from healthy and FCD patient-derived fibroblasts or peripheral blood mononuclear cells (PBMCs) with different somatic and germline mutations (pictured in different colors) to 3D cerebral organoids. These tools allow for the investigation of the excitation/inhibition (E/I) imbalance at a cellular level, already proposed as a cause of the molecular mechanisms of FCD. A key point is the dysregulation of chloride homeostasis, where an altered NKCC1/KCC2 ratio leads to persistent depolarizing GABAergic signaling expressed by immature/epileptic neurons. Another key point is the characterization of the neural lineage of FCD patients modeled using brain organoids. Future directions aim to increase model physiological relevance through dorso-ventral assembloids to assess deficits in the neuronal migration and integration, also incorporating non-neuronal lineages such as microglia and oligodendrocytes for a comprehensive understanding of FCD pathogenesis. The figure was formed with Biorender open source software.

By successfully overcoming these obstacles, these new technologies can serve as powerful platforms for studying neural network dysfunctions, such as those underlying FCDs, identifying the GABAergic switch through the calcium and chloride imaging, screening potential drug candidates, and developing personalized therapeutic strategies, as schematically illustrated in Figure 2 (Wang et al., 2025). Future efforts must prioritize enhancing their structural complexity, focusing on the generation of diverse connected regions, the formation of layered structures and of defined functional networks. This will be a crucial milestone to better understand the human brain specialization and the pathological mechanisms beneath it.
